# Decontamination of Chlorpyrifos Residue in Soil by Using *Mentha piperita* (Lamiales: Lamiaceae) for Phytoremediation and Two Bacterial Strains

**DOI:** 10.3390/toxics12060435

**Published:** 2024-06-16

**Authors:** Ahmed A. A. Aioub, Mohamed A. Fahmy, Esraa E. Ammar, Mohamed Maher, Heba A. Ismail, Jin Yue, Qichun Zhang, Sarah I. Z. Abdel-Wahab

**Affiliations:** 1 Zhejiang Provincial Key Laboratory of Agricultural Resources and Environment, Key Laboratory of Environment Remediation and Ecological Health, Zhejiang University, Ministry of Education, Hangzhou 310058, China; a.aioub@zu.edu.eg; 2 Plant Protection Department, Faculty of Agriculture, Zagazig University, Zagazig 44511, Egypt; sarahibrahimzaki88@gmail.com; 3 Department of Agricultural Microbiology, Faculty of Agriculture, Zagazig University, Zagazig 44511, Egypt; mohamed.microbiology1991@gmail.com; 4 School of Food and Biological Engineering, Jiangsu University, Zhenjiang 212013, China; esraa_ammar@science.tanta.edu.eg; 5 Plant Ecology Sector, Botany Department, Faculty of Science, Tanta University, Tanta 31527, Egypt; 6 Department of Biochemistry, Faculty of Agriculture, Zagazig University, Zagazig 44511, Egypt; 7 Plant Protection Research Institute, Agricultural Research Center, Dokki, Giza 12618, Egypt; heba.i.ma@hotmail.com; 8 Anji County Agriculture and Rural Bureau, Hangzhou 313300, China

**Keywords:** phytoremediation, chlorpyrifos, *Bacillus subtilis*, *Pseudomonas aeruginosa*, polluted soil

## Abstract

This study utilizes *Mentha piperita* (MI) for the first time to investigate the uptake and translocation of chlorpyrifos (CPF; 10 µg g^−1^) from soil, introducing a new approach to improve the efficacy of this technique, which includes using biosurfactants (*Bacillus subtilis* and *Pseudomonas aeruginosa*) at 10^7^ CFU/mL to degrade CPF under greenhouse conditions. Moreover, antioxidant enzymes, including superoxide dismutase (SOD) and peroxidase (Prx), and oxidative stress due to hydrogen peroxide (H_2_O_2_) and malondialdehyde (MDA) in MI roots and leaves were evaluated under CPF stress. Our results demonstrated that amending soil with MI and *B. subtilis* followed by *P. aeruginosa* significantly reduced CPF levels in the soil (*p* > 0.05) and enhanced CPF concentrations in MI roots and leaves after 1, 3, 7, 10, and 14 days of the experiment. Furthermore, CPF showed its longest half-life (t_1/2_) in soil contaminated solely with CPF, lasting 15.36 days. Conversely, its shortest half-life occurred in soil contaminated with CPF and treated with MI along with *B. subtilis*, lasting 4.65 days. Soil contaminated with CPF and treated with MI and *P. aeruginosa* showed a half-life of 7.98 days. The half-life (t_1/2_) of CPF-contaminated soil with MI alone was 11.41 days. A batch equilibrium technique showed that *B. subtilis* is better than *P. aeruginosa* for eliminating CPF from soil in In vitro experiments. Notably, CPF-polluted soil treated with coadministration of MI and the tested bacteria improved the activities of SOD and Prx and reduced H_2_O_2_ and MDA compared with CPF-polluted soil treated with MI alone. Our findings demonstrated that using *B. subtilis* and *P. aeruginosa* as biosurfactants to augment phytoremediation represents a commendable strategy for enhancing the remediation of CPF contamination in affected sites while reducing the existence of harmful pesticide remnants in crop plants.

## 1. Introduction

Chlorpyrifos (CPF), which exhibits an extensive variety of insecticidal properties against economically significant agricultural pests, is extensively utilized as an organophosphate insecticide on a global scale [1]. However, CPF presents potential hazards to non-target organisms, and its remnants endure in the environment for an indeterminate duration [2]. Moreover, vegetables cultivated in polluted regions represent a substantial hazard to human well-being [3]. The alleged toxicity of this substance to human beings is purportedly associated with a range of clinical symptoms, including brain dysfunction, endocrine disturbance, and cardiovascular illness [4]. Moreover, the half-life (t_1/2_) of CPF and its products in soil shows that they are persistent; depending on the soil conditions, deterioration typically takes 111 to 350 days [5]. It exhibits moderate toxicity, with low water solubility (0.73 mg/L at 25 °C) and high soil absorption (organic carbon partition coefficient log K_oc_ = 3.78 l/kg) [6]. The logarithm of the octanol–water partition coefficient (log K_ow_) for CPF is 5 [7]. The use of CPF in the United States is subject to limitations due to the presence of detrimental consequences [8]. In European Union nations, CPF has been banned due to its association with DNA damage triggered by oxidative stress or inhibition of topoisomerase II. These are regarded as molecular events linked to infant leukemia. Additionally, CPF has been classified as toxic for reproduction, posing risks to unborn children [9]. Nevertheless, its utilization remains prevalent in developing countries, such as Egypt, owing to its extensive applicability, cost-effectiveness, and notable efficacy [2]. Therefore, there is an urgent global need to reduce CPF residues in soil to eliminate their negative environmental impacts.

Several techniques, such as excavation, physical extraction, and in situ stabilization, are available to eliminate hazardous soil substances. Although they are highly effective, most of these methods have a significant cost [10]. Furthermore, besides these methods, the cultivation of plants can aid in the containment or mitigation of toxic pollutants. This process is commonly referred to as phytoremediation [10]. Phytoremediation involves utilizing plants to partially or significantly alleviate specific contaminants, including pesticides [11]. Phytoremediation is cost-effective, minimally disrupts soil structure, and enjoys greater public acceptance compared to alternative methods [12]. This method is currently extensively employed to cleanse organic or heavy metal pollutants in contaminated locations, such as agricultural lands, waste disposal sites, and polluted water bodies [13]. The absorption, storage, movement, and breakdown of small-scale pollutants by plants have been proposed as significant mechanisms in phytoremediation technology [14]. Phytoremediation leverages the unique capacity of plant root systems to selectively absorb contaminants and distribute them throughout the entire plant, aiding in their removal [15]. Several studies have focused on pesticide removal by phytoremediation [16,17,18]. Nevertheless, phytoremediation faces notable challenges, largely due to its time-consuming process and the inherent constraints of plants in effectively gathering significant amounts of pollutants while sustaining healthy growth under toxic conditions [19]. Additionally, plants may have limited ability to accumulate pollutants and can be highly susceptible to soil contamination levels that are too high [20]. To address this challenge, we employed bacteria capable of breaking down contaminants to prevent plants from experiencing the harmful effects of pollutants [21]. Utilizing plant–microbial strategies to enhance the phytoremediation process could prove effective, particularly in contamination cases with highly toxic and persistent organic pollutants [22]. Bacteria capable of degrading pollutants can aid plants in adapting to contaminated environments by detoxifying the soil by directly breaking organic contaminants into minerals [23]. Additionally, substances secreted by plants stimulate the proliferation and functionality of prospective bacteria that degrade pollutants in the vicinity of the root zone [24]. *Bacillus* sp. and *Pseudomonas* sp. are widely recognized bacterial groups employed in bioremediation processes [25,26]. These microbes gathered from polluted areas demonstrate enhanced capabilities in breaking down and utilizing pesticides [27]. *Bacillus subtilis* could biodegrade pendimethalin [28] and profenofos [29]. Another study showed that *B. subtilis* and *P. aeruginosa* isolated from the Beni Suef governorate, Egypt, enhanced the degradation of malathion [30]. Several research investigations have recorded the utilization of plant–microbe combinations for pollution remediation [11,31,32]. Nevertheless, there is still a scarcity of research focusing on pesticide removal using this method [33]. 

*Mentha piperita* (Lamiales: Lamiaceae; MI) is a fragrant herb commonly utilized in medicinal items, culinary preparations, beverages, teas, and cosmetic formulations [34]. Emerging from its root system, the plant exhibits rapid growth, with stems reaching heights of 40–130 cm. The stems, characterized by their red-purple hue, are extensively branched, while the elongated–oval leaves feature serrated edges and a light green coloration [35]. Azmat et al. [36] noted that roots play a significant role in accumulating compounds, as they are directly in contact with harmful chemicals in the soil and can carry these substances to the aboveground portions (leaves). Numerous research investigations have evaluated heavy metal remediation by MI [37,38,39,40]. To date, according to our knowledge, no data have been published about the role of MI in pesticide-polluted soil remediation.

Mounting evidence has suggested that the immoderate intracellular generation of reactive oxygen species (ROS) enables the determination of plant cell health [41]. Plants have developed different defense strategies to alleviate the harm induced by ROS. One such defense system is the enzymatic antioxidant mechanism, which comprises enzymes such as peroxidase (Prx) and superoxide dismutase (SOD). ROS generation is a popular event under stressful conditions and is influenced by enzymatic and nonenzymatic reactions [42]. Elevated ROS levels can activate antioxidant cascade pathways [43]. ROS-scavenging enzymes, such as SOD, promote the breakdown of hydrogen peroxide (H_2_O_2_) through dismutation [44]. Various other scavenging enzymes simultaneously convert H_2_O_2_ into water and oxygen [45]. Malondialdehyde (MDA) is generated as a final product when polyunsaturated fatty acids undergo decomposition during the peroxidation of membrane lipids. Its levels act as a marker for lipid peroxidation and oxidative harm [46]. The stress response to toxic substances typically involves ROS generation and lipid peroxidation [47].

This study hypothesized that the phytoremediation of *M. piperita* (MI) and microorganisms would be an excellent, inexpensive, and fast solution for cleaning polluted soil from CPF residue. Our findings aim to evaluate the potential of *M. piperita* (MI) and two bacterial strains (*Bacillus subtilis* and *Pseudomonas aeruginosa*) for the degradation and uptake of CPF-polluted soil under greenhouse conditions. Moreover, assessing the impact of CPF contamination on the enzymatic antioxidant system of MI roots and leaves involves analyzing the levels of SOD, Prx, MDA, and H_2_O_2_.

## 2. Materials and Methods

### 2.1. Insecticide and Bacteria

Chlorpyrifos (CPF) (97% purity) was obtained from the Agricultural Research Center, Egypt. The characteristics of CPF are outlined in Table 1. *Bacillus subtilis* subsp. subtilis AZFS3 (LC599401.1) and *Pseudomonas aeruginosa* KZFS4 (LC599404.1) were garnered from the Agricultural Microbiology Department, Faculty of Agriculture, Zagazig University, Egypt. Based on 16S rRNA gene sequencing, *B. subtilis* subsp. subtilis AZFS3 (LC599401.1) and *P. aeruginosa* KZFS4 (LC599404.1) were isolated from pesticide-contaminated soil, as presented in Fahmy et al. [48]. Moreover, antioxidant enzyme kits for Prx and SOD, as well as oxidative stress kits for MDA and H_2_O_2_, were sourced from the Biotechnology (SAE) Egyptian Co., Cairo, Egypt.

### 2.2. Phytoremediation Experiment Setup and Procedures

A greenhouse study was conducted under ambient light, with temperatures ranging from 25 to 27 °C, and humidity was maintained between 66% and 69% to evaluate the effects of MI with and without the tested bacteria on the uptake of the CPF residue from polluted soil. The soil utilized in the research was obtained from a location in Giza (28.7666° N, 29.2321° E), Egypt. The clay loamy soil, sifted through a sieve, was allowed to air-dry (organic matter = 1.82%; pH = 7.1; electric conductivity = 2.28 S m^−1^). Subsequently, it was transferred into plastic pots. Each plastic pot was filled with 500 g of soil. The *M. piperita* L. (MI) seeds were purchased from a nearby market in Giza, Egypt. The experimental pots were organized in a randomized layout of five distinct treatments: (1) unpolluted soil plus MI without CPF (S+MI); (2) CPF-polluted soil without MI (S+CPF); (3) CPF-polluted soil plus MI (S+MI+CPF); (4) CPF-polluted soil plus MI and *B. subtilis* at 10^7^ CFU/mL (S+MI+CPF+BS); (5) CPF-polluted soil plus MI with *P. aeruginosa* at 10^7^ CFU/mL (S+MI+CPF+PA). Each pot was planted with eight MI seeds. The MI plants were carefully thinned after germination to maintain only one plant per pot. Nonsterilized soil was used in the experiment to investigate how *B. subtilis* and *P. aeruginosa* interact with the indigenous microbial community in degrading CPF-contaminated soil under field-like conditions. In treatments 4 and 5, the MI seeds were permitted to grow for two weeks following inoculation with the tested bacteria at a concentration of 10^7^ CFU/mL (Tables S1 and S2). CPF was dissolved in 0.5 mL of acetone to obtain a concentration of 10 µg g^−1^. In the third week, treatments 2 to 5 received 10 µg g^−1^ of CPF. The MI plants were collected from the polluted soil for investigation at intervals of 1, 3, 7, 10, and 14 days following exposure to CPF. They were dissected, and their roots and leaves were separated. The MI roots underwent a two-minute wash under running tap water, followed by drying. The CPF residues were analyzed using 8 g of soil and 5 g of roots and leaves. The samples were preserved at −80 °C until subsequent analysis. In our study, a combined total of 90 pots were used for all treatments. Each treatment comprised 15 pots, with samples collected at five different time points, and three replicates were taken for each time point.

### 2.3. Biosurfactants Enhanced Recovery of CPF

In vitro research was conducted to assess the equilibrium adsorption isotherms of CPF, aiming to evaluate biosurfactants’ efficacy in enhancing CPF removal from polluted soil. Thus, 2 mL of two tested bacteria (*B. subtilis* and *P. aeruginosa*) at 10^7^ CFU/mL was separately added to 100 mL flasks. The control in this experiment was distilled water. This experiment was conducted with three replicates. Subsequently, CPF was introduced into all conical flasks at a concentration of 10 µg mL^−1^. The total volume of all treatments in each flask was adjusted to 20 mL. Then, 1 g of soil was added to the above treatments, and the samples were incubated with shaking for 3 h. Subsequently, the resultant suspensions were maintained for 24 h at 27 °C. The suspensions underwent centrifugation for 10 min at 15,000 rpm, after which CPF concentration in the supernatants was assessed using HPLC to quantify the amount of CPF present.

### 2.4. CPF Residue Extraction and Analysis in Soil and IM

The extraction of CPF from the samples was conducted utilizing the QuEChERS method outlined in [49]. Briefly, 8 g of experimental soil and 5 g of roots and leaves were put into a 50 mL centrifuge tube, and then 10 mL of acetonitrile containing 1% acetic acid was added. The samples underwent vigorous shaking for one minute, followed by the addition of 6 g of MgSO_4_, 1.5 g of NaCl, and 1 g of sodium citrate tribasic dihydrate. Each tube was shaken immediately after the salt was added. The tubes were vigorously shaken for 1 min and then centrifuged at 4000 rpm for 5 min. A 1 mL portion of the supernatant was transferred to a clean-up tube containing C18, MgSO_4_, primary secondary amine (PSA), and graphitized carbon black (GCB). The tubes were agitated for 30 s and subsequently centrifuged at 4000 rpm for 5 min.

### 2.5. HPLC Analysis

The amount of CPF residues in the collected samples was analyzed using an HPLC fitted with a UV detector. The HPLC system eluted isocratically with a mobile phase consisting of water and acetonitrile (70% acetonitrile + 30% water with 0.1% acetic acid). The flow rate was controlled at 1.5 mL per minute, and the total duration of the run was 15 min. A C18 column (Chiralpak IB- 250 mm × 4.6 mm, with a particle size of 5 µm) programmed at 25 °C was used. The eluents were monitored via UV detection with a wavelength equal to 280 nm. The retention time for CPF was recorded as 8.30 min.

### 2.6. Accuracy and Precision

The effectiveness of the HPLC method was evaluated by examining various parameters, including the values of recovery and the limits of quantification (LOQ) and detection (LOD). The linearity demonstrated significance, with an outstanding correlation coefficient of R = 0.996. The LOQ value was 0.62 µg g^−1^ and the LOD value was 0.14 µg g^−1^. CPF recoveries were evaluated at the fortification levels of 0.05, 0.1, and 0.5 mg kg^−1^ across the soil, root, and leaf samples. Under the specified conditions, no interfering peaks were observed on the chromatogram. The average recovery ranges for the soil, roots, and leaves were as follows: 93.5–95.3%, 88.12–90.70%, and 87.14–92.41%, respectively.

### 2.7. Physiological Parameters

A total of 100 mg of roots and leaves was collected from treatments 1, 3, 4, and 5 after 1, 3, 7, 10, and 14 days of CPF exposure and homogenized at 4 °C with liquid nitrogen and 100 mM phosphate buffer (pH 7.0) involving 1 mM PMSF, 0.5% PVP, and 1 mM EDTA. The blend was centrifuged at 9000 rpm for 20 min. The enzyme extract’s supernatant was used to evaluate the enzyme’s activity. SOD, Prx, MDA, and H_2_O_2_ kits were purchased from Biotechnology (SAE) Egyptian Co., Egypt.

### 2.8. Data and Statistical Analysis

One-way ANOVA was conducted, and the mean values (mean ± standard deviation) were compared across all treatments. CoStat 6.311 CoHort Statistical Software (https://cohortsoftware.com/costat.html, accessed on 1 June 2024) was employed for the study objectives. The significance level was set at *p* ≤ 0.05.

According to Romeh [50], the quantity of CPF adsorbed was determined by subtracting the initial concentration of CPF from the concentration at equilibrium.

x/m = (C_0_ − Ce) V/W. (1)

In this equation, x/m represents the concentration of CPF in the soil (µg g^−1^), C_0_ is the initial concentration of CPF (µg mL^−1^), Ce is the CPF concentration at equilibrium (µg mL^−1^), V denotes the solution volume, and W indicates the weight of the soil sample.

The determination of LOQ and LOD followed the equations according to European Commission guidelines for pesticide residue analytical methods [51]:

LOD = 3.3 S_0_/b and LOQ = 10 S_0_/b, (2)

where S_o_ represents the standard deviation of the calibration line and b denotes the slope.

The degradation rate (K) and half-life value (t_1/2_) were determined using the equations described by Gomaa and Belal [52] as follows:

The degradation rate (K) = 2.303 × slope.(3)

Half-life value (t_1/2_) = 0.693 K^−1^. (4)

## 3. Results

### 3.1. Degradation and Translocation of CPF under Tested Bacteria

**Figure 1:**
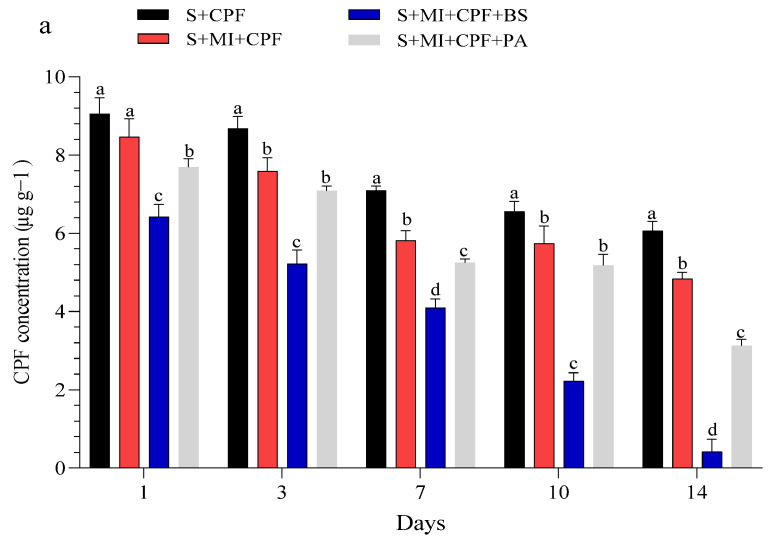

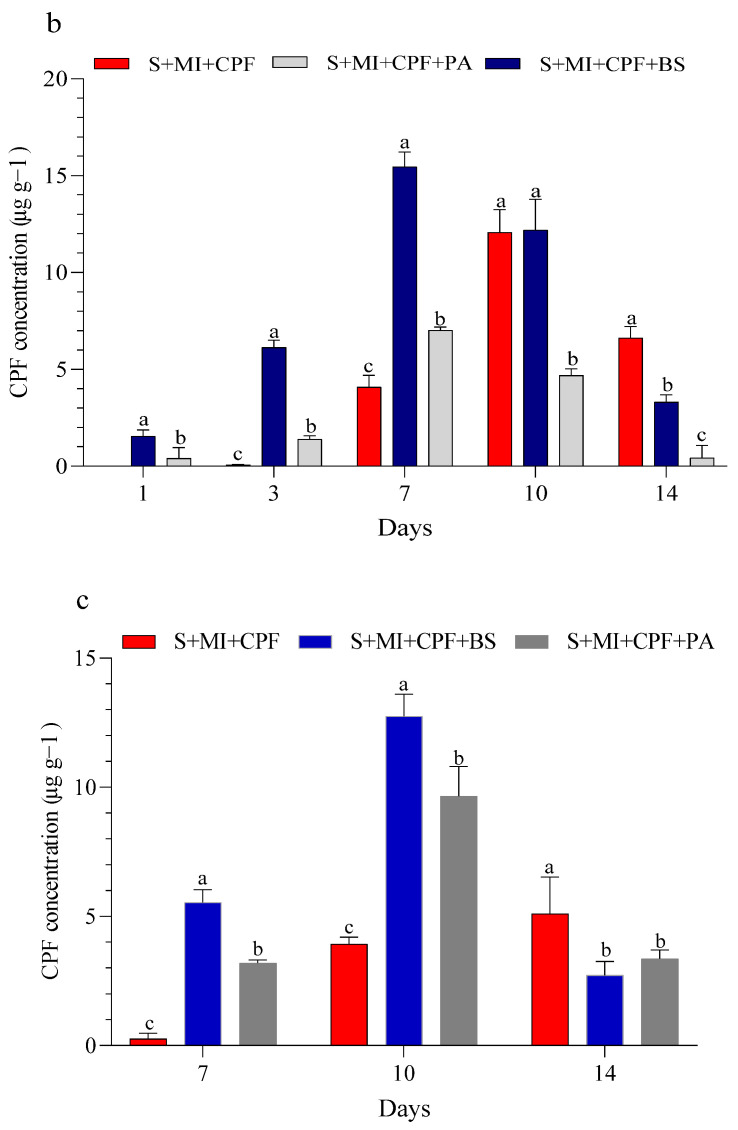
Efficacy of the two tested bacteria (*Bacillus subtilis* and *Pseudomonas aeruginosa*) in the phytoremediation of chlorpyrifos (CPF)-polluted soil using *Mentha piperita* (MI) during the 14 days of the experiment. CPF in soil (**a**), CPF in roots (**b**), and CPF in leaves (**c**). Means and standard deviations of three replicates. Different letters on top of the bar indicate significant differences (*p* < 0.05). S+CPF, CPF-polluted soil without MI; S+MI+CPF, CPF-polluted soil plus MI; S+MI+CPF+BS, CPF-polluted soil plus MI and *B. subtilis*; S+MI+CPF+PA, CPF-polluted soil plus MI and *P. aeruginosa*.

Figure 1 displays the CPF concentrations in the experimental soil and MI tissues throughout the study. Our findings indicated that all experimental groups effectively reduced a significant portion of CPF. Adding MI along with the tested bacteria to the soil reduced the CPF residues while simultaneously increasing the CPF concentrations in the roots and leaves of MI. *B. subtilis* and *P. aeruginosa* had a surfactant effect on the removal, absorption, and movement of CPF compared with MI alone. In the soil, the CPF concentration in the S+MI+CPF+BS treatment was in the range of 6.42–0.41 µg g^−1^ over the 14 days of the experiment, followed by the S+MI+CPF+PA treatment (7.69–3.12 µg g^−1^) and then 8.46–4.83 µg g^−1^ in the S+MI+CPF treatment compared with the S+CPF treatment (9.05–6.06 µg g^−1^) (Figure 1a). Concurrently, CPF remarkably accumulated in the MI roots to reach the highest levels after 7 days during the S+MI+CPF+BS treatment (15.46 µg g^−1^), followed by the S+MI+CPF+PA treatment (7.02 µg g^−1^) compared with the S+MI+CPF treatment. Conversely, no CPF concentration was observed in the leaves after 1 and 3 days of treatment. CPF moved into the MI leaves and reached 2.72 and 3.36 µg g^−1^ in the S+MI+CPF+BS and S+MI+CPF+PA treatments, respectively, after 14 days compared with the S+MI+CPF treatment (5.10 µg g^−1^) (Figure 1b,c).

### 3.2. Enhancing CPF Recovery in Soil

**Figure 2:**
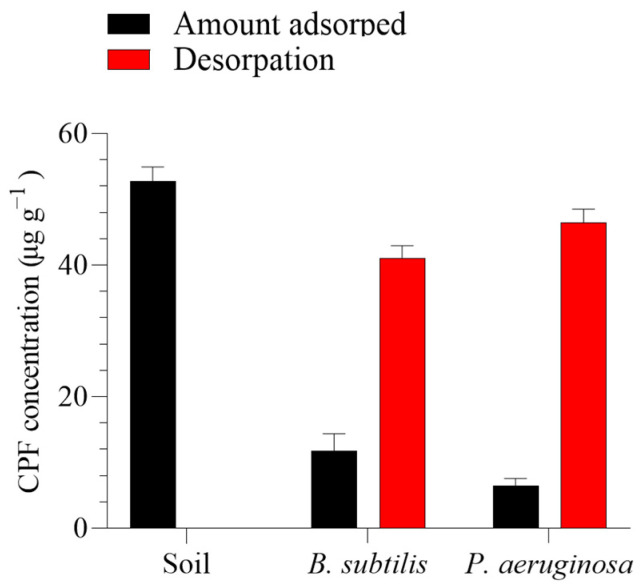
In vitro efficacy of the two tested bacteria (*Bacillus subtilis* and *Pseudomonas aeruginosa*) in improving chlorpyrifos (CPF) recovery from soil over 24 h. Means and standard deviations of three replicates.

As shown in Figure 2, a batch equilibrium method was employed to assess the capacity of *B. subtilis* and *P. aeruginosa* to adsorb CPF from the polluted soil by comparing the degradation-improving biosurfactants with the control. *B. subtilis* was significantly effective (*p* > 0.05) in reducing CPF adsorption onto the soils, followed by *P. aeruginosa*. The amount of CPF adsorbed by *B. subtilis* and *P. aeruginosa* from the polluted soil was 11.83 and 6.44 µg g^−1^, respectively, after one day compared to the soil’s adsorption of 52.84 µg g^−1^.

### 3.3. Reaction Rate Constants for CPF

**Figure 3:**
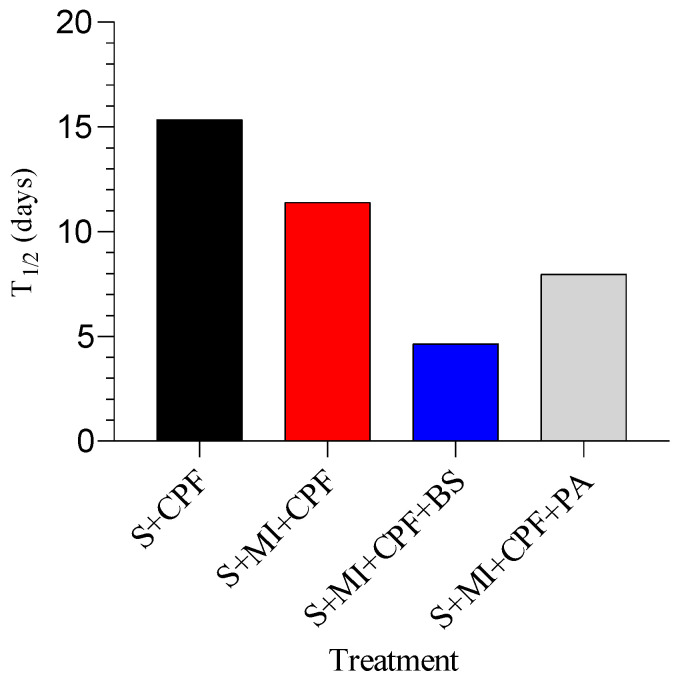
The half-lives (t_1/2_) of chlorpyrifos (CPF) under different soil treatments. S+CPF, CPF-polluted soil without MI; S+MI+CPF, CPF-polluted soil plus MI; S+MI+CPF+BS, CPF-polluted soil plus MI and *B. subtilis*; S+MI+CPF+PA, CPF-polluted soil plus MI and *P. aeruginosa*.

Using a first-order reaction, T_1/2_ of CPF in the soil inoculated with MI along with *B. subtilis*, MI inoculated with *P. aeruginosa*, and MI alone was found to be 4.65, 7.98, and 11.41 days, respectively, compared to 15.36 days for the soil alone (Figure 3).

### 3.4. Physiological Characteristics of Mentha Piperita

**Figure 4:**
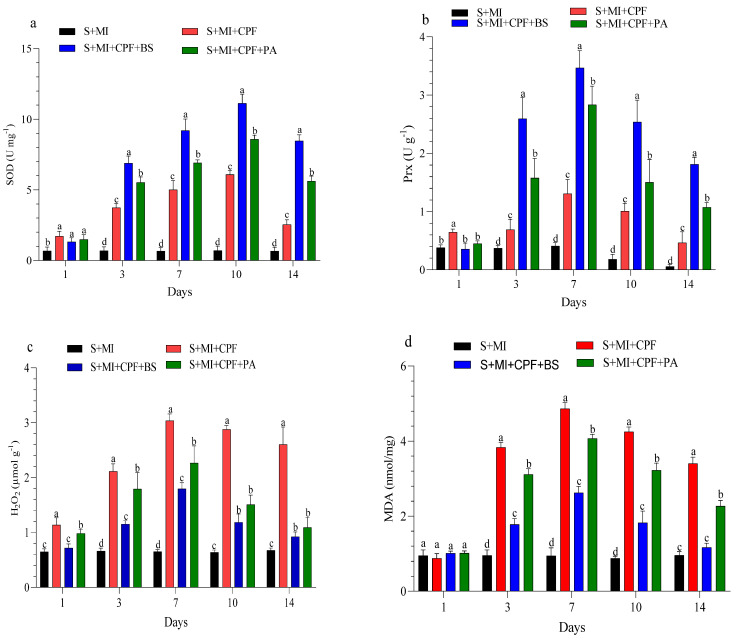
Levels of superoxide dismutase (SOD) (**a**), peroxidase (Prx) (**b**), hydrogen peroxide (H_2_O_2_) (**c**), and malondialdehyde (MDA) (**d**) in *Mentha piperita* (MI) roots inoculated with *Bacillus subtilis* and *Pseudomonas aeruginosa* in soil contaminated with chlorpyrifos (CPF) through 1–14 days of exposure. Means and standard deviations of three replicates. Different letters on top of the bar indicate significant differences (*p* < 0.05). S+CPF, CPF-polluted soil without MI; S+MI+CPF, CPF-polluted soil plus MI; S+MI+CPF+BS, CPF-polluted soil plus MI and *B. subtilis*; S+MI+CPF+PA, CPF-polluted soil plus MI and *P. aeruginosa*.

**Figure 5:**
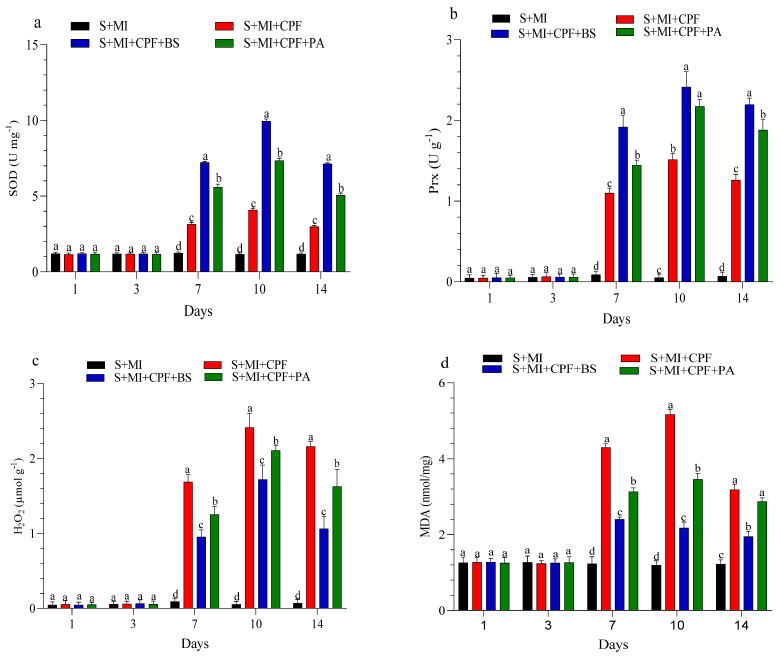
Levels of superoxide dismutase (SOD) (**a**), peroxidase (Prx) (**b**), hydrogen peroxide (H_2_O_2_) (**c**), and malondialdehyde (MDA) (**d**) in *Mentha piperita* (MI) leaves inoculated with *Bacillus subtilis* and *Pseudomonas aeruginosa* in soil contaminated with chlorpyrifos (CPF) through 1–14 days of exposure. Means and standard deviations of three replicates. Different letters on top of the bar indicate significant differences (*p* < 0.05). S+CPF, CPF-polluted soil without MI; S+MI+CPF, CPF-polluted soil plus MI; S+MI+CPF+BS, CPF-polluted soil plus MI and *B. subtilis*; S+MI+CPF+PA, CPF-polluted soil plus MI and *P. aeruginosa*.

The changes in enzyme activity and oxidative enzymes in the roots and leaves of MI after 1, 3, 7, 10, and 14 days are shown in Figure 4 and Figure 5. MI amended with *B. subtilis* and *P. aeruginosa* under CPF stress in the roots and leaves significantly enhanced SOD and Prx activity compared with the S+MI+CPF treatment. SOD activity in the S+MI+CPF+BS and S+MI+CPF+PA treatments was achieved after 10 days of CPF exposure in the roots (11.1 and 8.58 U g^−1^) and leaves (9.95 and 7.34 U g^−1^) compared with S+MI+CPF alone (6.09 and 4.08 U g^−1^), respectively. The Prx activity in the roots under CPF stress after 7 and 14 days was recorded (*p* > 0.05) at 3.46 and 1.81 U g^−1^ in the S+MI+CPF+BS treatment and at 2.83 and 1.07 U g^−1^ in the S+MI+CPF+PA treatment compared with the S+MI+CPF treatment (1.30 and 0.46 U g^−1^), respectively. No significant difference was observed in the Prx activity of the MI leaves among all treatments after 1 and 3 days of CPF exposure. Meanwhile, the Prx activity of the MI leaves increased in the S+MI+CPF+BS treatment, followed by the S+MI+CPF+PA treatment compared with the S+MI+CPF treatment after 7 to 14 days of CPF exposure.

H_2_O_2_ content remarkably decreased (*p* > 0.05) in the MI roots and leaves amended with *B. subtilis* and *P. aeruginosa* compared with MI alone under CPF stress. H_2_O_2_ content reached the highest value after 7 days in the MI roots and after 10 days in the MI leaves during the S+MI+CPF treatment (3.03 and 2.41 µmol g^−1^) compared with the S+MI+CPF+BS treatment (1.79 and 1.72 µmol g^−1^) and the S+MI+CPF+PA treatment (2.26 and 2.10 µmol g^−1^), respectively. MDA content in the MI roots and leaves was 4.86 and 4.30 nmol/mg during the S+MI+CPF treatment, followed by the S+MI+CPF+PA treatment (4.07 and 3.13 nmol/mg) and then the S+MI+CPF+BA treatment (2.62 and 2.40 nmol/mg) after 7 days of exposure, respectively.

## 4. Discussion

Environmental pollution on a global scale is one of the foremost environmental challenges facing modern society [53]. Phytoremediation, an emerging technology, has garnered attention for its potential to remediate polluted soil due to its cost-effectiveness, aesthetic benefits, and long-term viability [54]. According to our findings, MI can absorb CPF from polluted soil, significantly improving CPF removal from the soil. This capability stems from the MI root system’s high capacity for binding and accumulating xenobiotics, thus safeguarding the plant against pollutant toxicity [55,56]. Also, analytical quantification demonstrates that MI is suitable for remediating heavily contaminated soils [57]. Our findings align with those of Dinu et al. [58], who showed that MI can effectively stabilize metals at the root level and tolerate metals when cultivated in a nutrient-rich substrate. Moreover, MI has been recognized as a hyperaccumulator plant and is recommended for remediation efforts targeting heavy metals [59,60]. Furthermore, some factors affect the pesticide sorbed to the soil and translocation in plant tissues, such as soil types, pesticide (lipophilicity or hydrophilic), water solubility, log Koc, and log Kow values [61]. Turgut [62] found that the absorption and movement of organic compounds relies on factors such as hydrophobicity (lipophilicity), solubility, polarity, molecular weight, plant species, and environmental conditions. Therefore, according to our study, the absorption of CPF in soil and its movement in MI roots and leaves may be because of the physical and chemical properties of CPF, which shows lipophilicity with a low water solubility of 0.73 mg/L, as well as high log Koc and log Kow values, of 3.78 and 5 l/kg, respectively [7]. Bouldin et al. [63] recorded that the main characteristic determining the movement of pesticides such as CPF within plants is their lipophilicity, which correlated with the Kow value. Notably, pesticides should have a log Kow ranging between 3.0 and 0.5 to achieve optimal uptake. Pesticides with lower log Kow values are frequently too hydrophilic to penetrate the cell membrane, while those with higher log Kow values tend to be highly hydrophobic and can adhere strongly to roots [63]. In addition, when pesticides have a high Koc value, they are strongly absorbed by soil, making it challenging for plants to uptake and transport them [61]. Chlorpyrifos (CPF) has been predicted to penetrate biomembranes and subsequently bind to the roots [64], with minimal uptake and translocation to the aboveground biomass of plants. Foliar absorption directly into the aboveground parts of plants is a significant pathway, particularly for volatile and semi-volatile compounds, in comparison to root uptake [65]. Nonetheless, the volatility of CPF in soil was notably reduced over extended exposure periods [66]; consequently, CPF residue tended to accumulate more in the roots than in the aerial parts of the plant. Interestingly, when CPF is tightly bound to soil, its availability for microbial degradation and plant absorption is reduced [67]. Additionally, CPF exhibits strong binding to soil and remains immobile to slightly mobile within soil due to its low water solubility and high Koc value [68].

Amending soil with MI plus *B. subtilis* and *P. aeruginosa* reduced CPF in the soil from 1 to 14 days of the experiment, accompanied by enhancement in the plant leaves and roots. Applying *B. subtilis* and *P. aeruginosa* to polluted soil using a batch equilibrium technique demonstrated more effective removal of CPF than soil alone. This effect was shown by the microbial degradation mechanism, which can be outlined in three phases [69]. Initially, the focus was on adsorption, as CPF adhered to the cell membrane surface in a dynamic equilibrium process, which was crucial. Next, the compound entered the cell membrane surface, and its penetration efficiency and rate were affected by its molecular structure. Lastly, the compound undergoes rapid enzymatic reactions within the membrane. As previously demonstrated by Gongora-Echeverria et al. [70], the bioremediation of bacteria proves effective in detoxifying accumulated pesticide residues in the environment. Furthermore, using *B. subtilis* culture holds promising potential for remedying agricultural soils contaminated with monocrotophos [71] and glyphosate [72]. Moreover, *P. aeruginosa* can degrade CPF, cypermethrin, and endosulfan [73,74]. Likewise, *B. subtilis* and *P. aeruginosa* have biodegradation properties that metabolize chlorantraniliprole-, flubendiamide-, and cypermethrin-contaminated soil [48,75,76]. Singh [77] advocated for the inoculation of soil with *Pseudomonas* sp., which accelerated CPF degradation in Australia. Interestingly, the success of bioaugmentation relies heavily on the inoculum density because introducing bacteria into the environment has a competitive advantage over native bacteria in exploiting space and nutrients [78]. The rate and efficiency of pesticide degradation are positively correlated with the inoculum density [79]: higher inoculum densities provide resilience against some environmental factors such as soil type and pH [80,81], and increasing the inoculum density can enhance the overall biodegradation capacity of the microbial community [82]. For example, the authors reported that a high inoculum density (>10^6^ cells/g of soil) significantly influences the rate at which CPF degrades [80]. Singh et al. [83] demonstrated that *Enterobacter* sp. shows no degradation of CPF when introduced into soil at an inoculum density below 10^3^ cells/g of soil. Moreover, the degradation rate of CPF in soil inoculated with *Stenotrophomonas* sp. was enhanced as soil pH increased from 4.3 to 7. However, there was no notable difference in the degradation rate between soil pH of 7 and 8.4 [84]. Also, introducing diazinon-degrading *S. marcescens* into soils accelerated the degradation of CPF, reducing its half-life (T1/2) by 20.7, 11.9, and 9.7 days in sandy, sandy loam, and silty soils, respectively, compared to soils without the inoculum [85]. Microbe-assisted phytoremediation holds significant promise for remediating soil polluted with pesticides [11,86]. MI does not possess the capability of degrading CPF; instead, it can only absorb CPF-polluted soil. However, soil microbes are essential for CPF degradation. In soil alone, the T_1/2_ value of CPF was recorded at 15.3 days. In contrast, in soil amended with *B. subtilis*, it was significantly shorter, at 4.65 days. The improvement in CPF dissipation within the phytoremediation system is attributed to the decrease in its half-life, likely resulting from CPF sorption to the plant metabolism, or improved degradation facilitated by the collaborative impact of plants and microorganisms in the root zone [11]. This study aligns with the findings of Malla et al. [87], who found that the T_1/2_ value for CPF degradation by *Bacillus cereus* in polluted soil was 1.26 days. Another study showed that the T_1/2_ of CPF was 3.01 days in *Brassica oleracea* and 1.35 days in *Brassica nigra* leaves [88].

Multiple findings have indicated that plants have developed a sophisticated antioxidant defense mechanism relying on SOD and Prx to combat free radicals’ action [89]. Enhanced stress tolerance in plants exposed to diverse stressors is linked to improved antioxidant enzyme activity [90]. Our findings showed that antioxidant activity (SOD and Prx) in the root and leaves of MI remarkably increased and oxidative stress (MDA and H_2_O_2_) decreased in the presence of the two tested bacteria compared with MI alone. An enhancement in the activity of SOD and Prx may be due to SOD enzymes playing a crucial role at the forefront of defense against ROS. Prx participates in the detoxification of H_2_O_2_. Notably, Prx can catalyze hydroxylic reactions as a secondary cyclic reaction, distinct from peroxidation reactions. Additionally, Prx participates in the breakdown of H_2_O_2_ [91]. The reduction in lipid peroxidation observed with *B. subtilis* and *P. aeruginosa* inoculation under CPF stress could be attributed to the increased synthesis of ROS-scavenging enzymes. Interestingly, xenobiotic-induced lipid peroxidation occurs because of the elimination of hydrogen from fatty acids by ROS, resulting in the generation of lipid radicals [92]. This initiates a reaction cascade, forming short-chain alkanes and acidic aldehydes, which disrupt the lipid structure [93]. These findings align with those of prior research demonstrating that bacterial inoculation enhances plant tolerance to xenobiotics by enhancing the antioxidative activity of various enzymes [94,95]. Gururani et al. [96] found *Bacillus pumilus* strain DH-11 and *Bacillus firmus* strain 40, which were isolated from the potato rhizosphere. These strains enhanced the zinc tolerance of potato plants by increasing the transcription levels of ROS-scavenging enzymes (Prx and SOD), thereby improving the plants’ tolerance to Zn. Furthermore, Martins et al. [97] showed that bacteria isolated from soil samples exhibit increased production of antioxidants in response to pesticide stress, such as acetochlor and metolachlor, suggesting that antioxidants serve as a mechanism for tolerance against oxidative stress. *Helianthus annuus* incubated with *Bacillus* sp. plus endosulfan decreased MDA and H_2_O_2_ production [98]. The inoculation of *Bacillus siamensis* in *Triticum aestivum* resulted in a reduction of cadmium toxicity, as evidenced by decreased MDA content and enhanced SOD levels [99]. Also, *Bacillus subtilis* decreased the MDA amount and enhanced *Medicago sativa* antioxidant enzyme activity under Cd stress [100]. Coadministration of PGPR and maize contaminated with Cd significantly reduced MDA and H_2_O_2_ content, restoring normal plant reactions [101].

## 5. Conclusions

Our study suggests that collaboration between plants and bacteria represents a promising new strategy for remediating chlorpyrifos (CPF)-contaminated soil. *Mentha piperita* (MI) can effectively extract CPF from polluted soil through its roots and move it to its leaves. Therefore, MI could be a promising model for mitigating CPF levels in contaminated soil. Moreover, *Bacillus subtilis* and *Pseudomonas aeruginosa* can improve the phytoremediation of CPF in polluted soil by serving as biosurfactants. Furthermore, antioxidant activity (SOD and Prx) was enhanced and oxidative stress (H_2_O_2_ and MDA) was reduced in the CPF-polluted soil treated with coadministration of MI and the tested bacteria compared with CPF-polluted soil treated with MI alone. Therefore, collaboration between phytoremediation and bacteria is suggested as a practical approach to expedite the elimination of pesticide residues from polluted soil, thereby ensuring the safety of both humans and non-target organisms.

## Figures and Tables

**Table 1:** Physical and chemical properties of CPF, according to Mackay, Giesy, and Solomon [7].

Molecular weight	350.6 g mol^−1^
Vapor pressure (25 °C)	1.87 × 10^−5^ mmHg
Water solubility (20 °C)	0.73 mg L^−1^
Henry’s law constant	1.10 × 10^−5^ atm m^−3^ mol^−1^
Log K_OW_	5
Log K_Oc_	3.78–3.93 L/kg

## Data Availability

All data and materials are included in the manuscript.

## Supplementary Materials

The following supporting information can be downloaded at: https://www.mdpi.com/article/10.3390/toxics12060435/s1, Table S1: Total bacterial count of the soil before being treated with CPF. Table S2: Total bacterial count of soil during the time course of the experiments.
