# Supplementary material for: Decontamination of Chlorpyrifos Residue in Soil by Using Mentha piperita (Lamiales: Lamiaceae) for Phytoremediation and Two Bacterial Strains
Source: Toxics. 2024 Jun 16;12(6):435. doi: 10.3390/toxics12060435 (PMC11209611; doi:10.3390/toxics12060435)
Supplement: Supplementary file 1 [file toxics-12-00435-s001.zip › toxics-3035520-supplementary.pdf]

Table S1. Total bacterial count of the soil before being treated with CPF

Treatment	Total bacterial counts (10^8 × CFU/g.d. wt. soil)	
	The first week of bacteria inoculation	The second week of bacteria inoculation
S+MI	0.11^c	0.12^c
S+MI+BS	1.30^a	1.28^a
S+MI+PA	1.21^b	1.18^b

Different letters represent significant differences (Duncan's test significant difference test at $p < 0.05$) among all treatments.

S: Soil, MI: *Mentha piperita*, BS: *Bacillus. Subtilis, PA: Pseudomonas aeruginosa*

Table S2. Total bacterial count of soil during the time course of experiments

Treatment	Total bacterial counts (10^8 × CFU/g.d. wt. soil)				
	**1 day**	**3 days**	**7 days**	**10 days**	**14 days**
S+MI	0.11^e	0.10^e	0.12^e	0.10^e	0.11^e
S+CPF	0.15^d	0.19^d	0.20^d	0.50^d	0.30^d
S+MI+CPF	0.17^c	0.30^c	0.76^c	1.11^c	0.95^c
S+MI+CPF+BS	1.33^a	1.55^a	1.88^a	2.38^a	1.89^a
S+MI+CPF+PA	1.23^b	1.42^b	1.55^b	2.11^b	1.66^b

Different letters represent significant differences (Duncan's test significant difference test at $p < 0.05$) among all treatments.

S: Soil, MI: *Mentha piperita*, BS: *Bacillus. Subtilis, PA: Pseudomonas aeruginosa*, CPF: Chlorpyrifos
